# Rogue AI: Cautionary Cases in Neuroradiology and What We Can Learn From Them

**DOI:** 10.7759/cureus.56317

**Published:** 2024-03-17

**Authors:** Austin Young, Kevin Tan, Faiq Tariq, Michael X Jin, Avraham Y Bluestone

**Affiliations:** 1 Department of Radiology, Stony Brook University Hospital, Stony Brook, USA

**Keywords:** ct head angiography, medical imaging, neuroradiology, radiology, ai, machine learning, artificial intelligence

## Abstract

Introduction

In recent years, artificial intelligence (AI) in medical imaging has undergone unprecedented innovation and advancement, sparking a revolutionary transformation in healthcare. The field of radiology is particularly implicated, as clinical radiologists are expected to interpret an ever-increasing number of complex cases in record time. Machine learning software purchased by our institution is expected to help our radiologists come to a more prompt diagnosis by delivering point-of-care quantitative analysis of suspicious findings and streamlining clinical workflow. This paper explores AI’s impact on neuroradiology, an area accounting for a substantial portion of recent radiology studies. We present a case series evaluating an AI software’s performance in detecting neurovascular findings, highlighting five cases where AI interpretations differed from radiologists’ assessments. Our study underscores common pitfalls of AI in the context of CT head angiograms, aiming to guide future AI algorithms.

Methods

We conducted a retrospective case series study at Stony Brook University Hospital, a large medical center in Stony Brook, New York, spanning from October 1, 2021 to December 31, 2021, analyzing 140 randomly sampled CT angiograms using AI software. This software assessed various neurovascular parameters, and AI findings were compared with neuroradiologists’ interpretations. Five cases with divergent interpretations were selected for detailed analysis.

Results

Five representative cases in which AI findings were discordant with radiologists’ interpretations are presented with diagnoses including diffuse anoxic ischemic injury, cortical laminar necrosis, colloid cyst, right superficial temporal artery‐to‐middle cerebral artery (STA‐MCA) bypass, and subacute bilateral subdural hematomas.

Discussion

The errors identified in our case series expose AI’s limitations in radiology. Our case series reveals that AI’s incorrect interpretations can stem from complexities in pathology, challenges in distinguishing densities, inability to identify artifacts, identifying post-surgical changes in normal anatomy, sensitivity limitations, and insufficient pattern recognition. AI’s potential for improvement lies in refining its algorithms to effectively recognize and differentiate pathologies. Incorporating more diverse training datasets, multimodal data, deep-reinforcement learning, clinical context, and real-time learning capabilities are some ways to improve AI’s performance in the field of radiology.

Conclusion

Overall, it is apparent that AI applications in radiology have much room for improvement before becoming more widely integrated into clinical workflows. While AI demonstrates remarkable potential to aid in diagnosis and streamline workflows, our case series highlights common pitfalls that underscore the need for continuous improvement. By refining algorithms, incorporating diverse datasets, embracing multimodal information, and leveraging innovative machine learning strategies, AI’s diagnostic accuracy can be significantly improved.

## Introduction

In recent years, the realm of artificial intelligence (AI) has undergone unprecedented innovation and advancement, sparking a revolutionary transformation in healthcare [1]. These breakthroughs have unlocked numerous opportunities for improving patient care and transforming the medical profession as a whole [1]. Within this rapidly evolving landscape, the field of radiology has witnessed a paradigm shift with the integration of AI into various aspects of medical imaging [2]. With advancements in graphics processing computer power and the availability of large datasets, significant strides have been made in computer-aided detection/diagnosis, disease prediction, and image segmentation, with some AI algorithms demonstrating remarkable performance matching that of radiologists [3]. Today, clinical radiologists are expected to interpret an ever-increasing number of complex cases in record time [4,5]. Machine learning software purchased by our institution is expected to help our radiologists come to a more prompt diagnosis by delivering point-of-care quantitative analysis of suspicious findings and streamlining clinical workflow.

One area within radiology where AI’s impact is particularly pronounced is neuroradiology, accounting for nearly one-third of radiology AI studies in recent years [6]. In this context, we showcase a pilot retrospective study to assess the performance of an AI software designed to detect various neurovascular findings recently acquired by our institution. We selected five cases where the interpretation conflicted with our radiologists’ interpretations. As we evaluate these cases, an emphasis will be placed on elucidating the common pitfalls of AI in neuroradiology particularly in the context of CT head angiograms, with the goal of providing direction for future deep learning algorithms.

## Materials and methods

We conducted a pilot retrospective case study to examine a random sample of CT head angiograms (n = 140) at Stony Brook University Hospital, a large medical center in Stony Brook, New York, spanning from October 1, 2021 to December 31, 2021. During this period, a total of 140 head CT angiograms were randomly sampled, and the images were subsequently subjected to analysis using RapidAI, a Federal Drug Administration-approved neuroimaging software. Inclusion criteria were all adult patients (aged 18 years and older) who underwent a CT head angiogram for various clinical indications. Exclusion criteria included poor image quality that precluded accurate analysis. The CT angiograms were then de-identified to maintain patient confidentiality and subjected to analysis by the AI software. The AI software assessed a spectrum of neurovascular parameters, including blood vessel density, large vessel occlusion, acute hemorrhage, and perfusion deficits to generate findings. The AI-generated findings were then compared with the findings of our experienced neuroradiologists, with over 20 years of experience. The attending-derived findings served as the gold standard for comparison and were obtained through a comprehensive chart review process. The chart review process included a detailed examination of the radiologist report that was available for each case.

An array of cases where AI findings and radiologist interpretations diverged were identified. A preliminary statistical analysis focused on the frequency of discordant cases across the specified neurovascular parameters was performed. This included calculating the percentage of cases that were in agreement with the AI-generated findings and the percentage of cases that were not in agreement. The cases that were not in agreement were further categorized into the neurovascular parameters assessed by the AI software. From the pool of cases, we selected five discordant cases for detailed analysis. A root cause analysis was conducted to identify the AI error and uncover the reasons behind the errors and their potential implications. This analysis aimed to dissect the nuances of each discordant case, pinpointing the specific factors contributing to the discrepancies observed. To achieve this, an in-depth discussion with a panel of experienced neuroradiologists was conducted. 

Statistical analysis

It is important to note that our assessment was primarily qualitative in nature, with only minimal statistical analysis conducted. The statistical analysis of divergent findings was conducted using descriptive statistics. Specifically, we calculated the percentages of concordant and discordant cases to quantify the agreement and discrepancies between the two sets of findings. In addition, it is important to approach the statistical analysis presented in this study with caution, as this research is based on a pilot study with a limited sample size.

## Results

A total of 140 CT head angiography (CTA) cases were analyzed by four radiologists over a three-month period. Of the 140 cases, 101 cases (72%) showed complete agreement between the attending radiologist's impressions and the AI analysis, indicating no discordance. However, in 39 cases (28%), there was some level of discordance observed across the four categories analyzed by the AI, namely, in the blood vessel density, large vessel occlusion, bleeding, and perfusion deficits. It is worth noting that some cases had multiple errors across those categories. Eighteen cases had a discordance with blood vessel density analysis. Fourteen cases had a discordance with the large vessel occlusion analysis. Twelve cases had a discordance with the bleeding analysis. Four cases had a discordance with the perfusion analysis.

Analysis of the 39 discordant reads revealed several common pitfalls of AI in the interpretation of CT head angiograms. Five common cases in which AI findings were discordant with radiologists’ interpretations are presented with diagnoses, including diffuse anoxic ischemic injury, cortical laminar necrosis, colloid cyst, right STA-MCA bypass, and subacute bilateral subdural hematomas.

Case 1: diffuse anoxic ischemic injury

**Figure 1:**
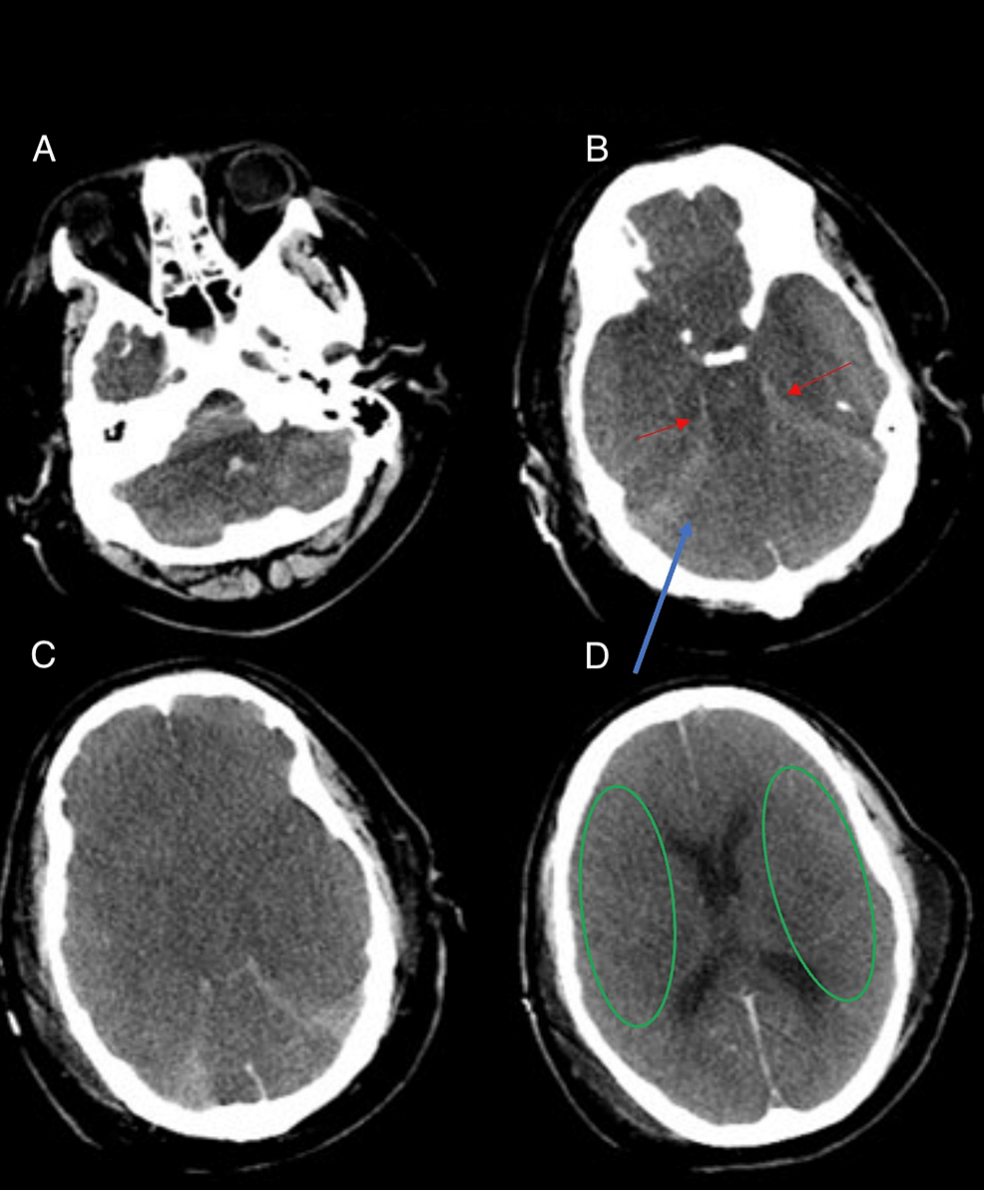
Four axial head images from a non-contrast head CT in a patient with diffuse anoxic ischemic injury. Panels A and C represent prior images taken for comparison. Panel B demonstrates a hyperdense area in the basilar cisterns (red arrows) and cerebellum (blue arrow). Panel D shows surrounding areas of diffuse sulcal effacement secondary to the anoxia (green circles).

Diffuse anoxic ischemic injury occurs in anoxic events in which the cerebral cortex is deprived of oxygen. Multiple axial images taken from non-contrast head CT demonstrate a hyperdense appearance of the basilar cisterns (red arrows), hyperdense cerebellum (blue arrows), and diffuse sulcal effacement (green circles) (Figure 1). This is characteristic of a diffuse anoxic ischemic injury. The AI mistakenly interpreted this as a subarachnoid hemorrhage (SAH) and subdural hemorrhage (SDH). 

Case 2: cortical laminar necrosis

**Figure 2:**
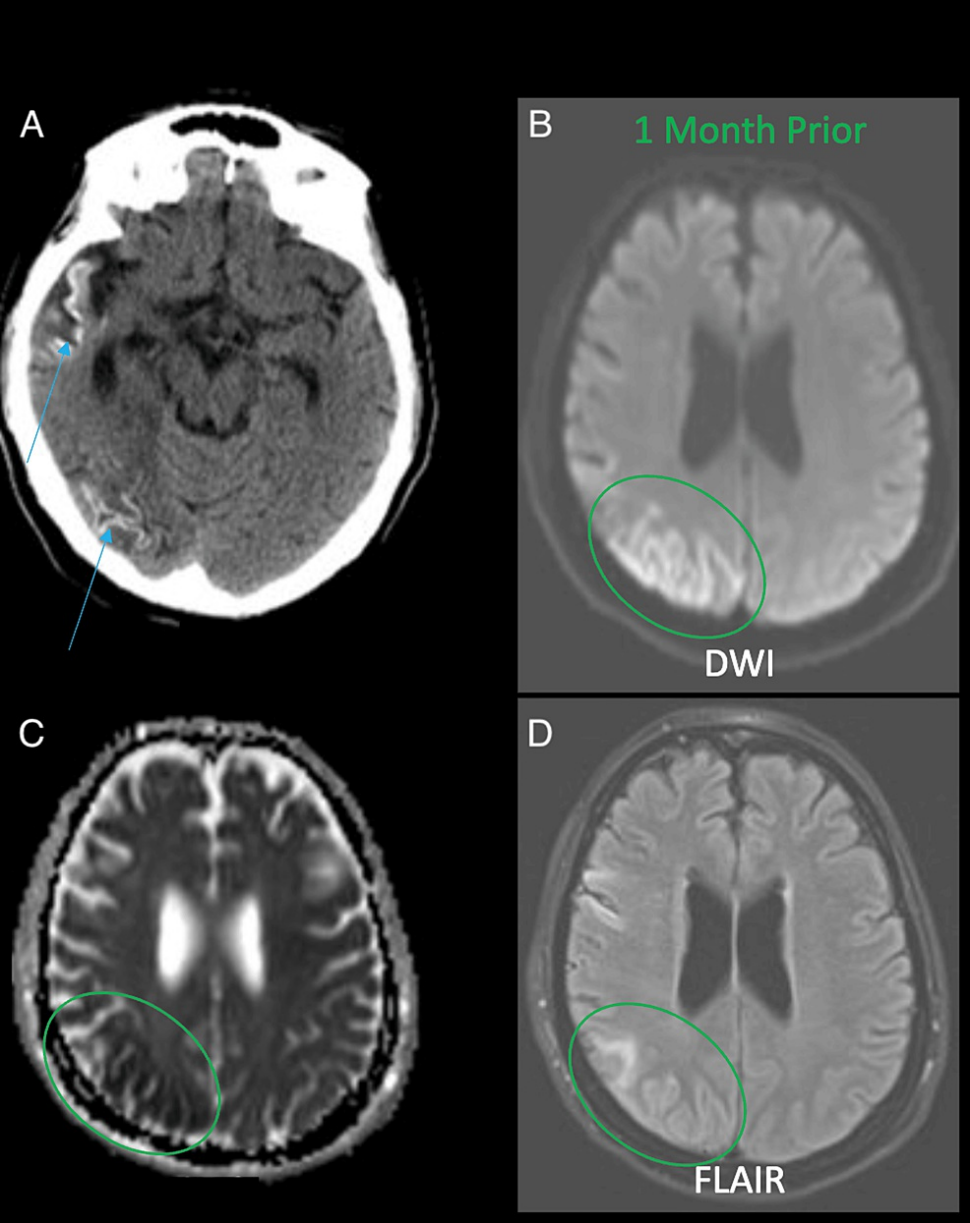
Two sets of images, CT (left) and MRI (right) in a patient one month apart demonstrating cortical laminar necrosis. Panel A shows areas of increased laminar density secondary to cortical laminar necrosis (blue arrow). Panel C highlights the same area of laminar calcification (green circle). Panels B and D demonstrate an area of decreased diffusion with increased DWI and decreased ADC indicating an acute infarction, due to cortical laminar necrosis (green circle). DWI: diffusion-weighted image, ADC: apparent diffusion coefficient

Cortical laminar necrosis, also known as pseudolaminar necrosis, is the end stage of cell death from injury to the brain. There is increased density on CT (top-left image) due to cortical laminar necrosis (blue arrows) (Figure 2). The MRI one month prior demonstrated restricted diffusion (increased DWI and decreased ADC) with fluid-attenuated inversion recovery (FLAIR) hyperintensity consistent with an acute infarction in the same distribution. As such, this represents a right parietal and temporal cortical laminar necrosis on CT secondary to evolving subacute to chronic infarction. The AI incorrectly identified the increased density in the right parietal and temporal regions as apparent acute hemorrhage. 

Case 3: colloid cyst

**Figure 3:**
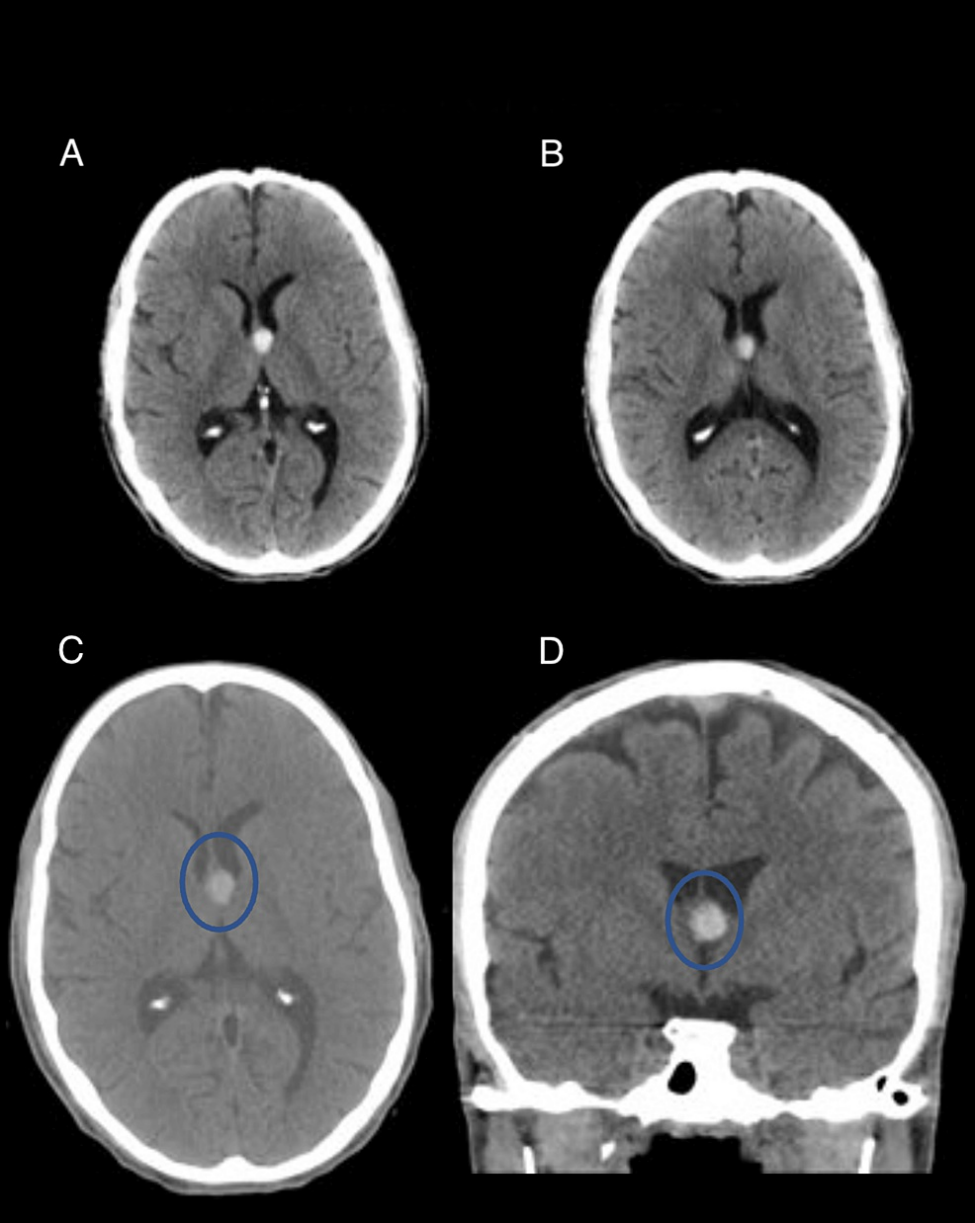
Four axial slices of a non-contrast head CT in a patient with a benign colloid cyst. Panels C and D highlight the benign protein-filled hyperdensity present in the Foramen of Monro (blue circles).

Colloid cysts are benign brain lesions filled with a proteinaceous substance called colloid, typically found within the fluid-filled ventricles of the brain. It is characteristically found on the roof of the third ventricle near the foramen of Monro. Figure 3 shows a well-circumscribed hyperdense lesion at the foramen of Monro (blue circle). This lesion is found at the classic location and with the density characteristic of a colloid cyst. Colloid cysts typically exhibit this hyperdensity due to their highly proteinaceous content. The AI incorrectly interpreted the lesion as an acute hemorrhage. 

Case 4: right STA-MCA bypass

**Figure 4:**
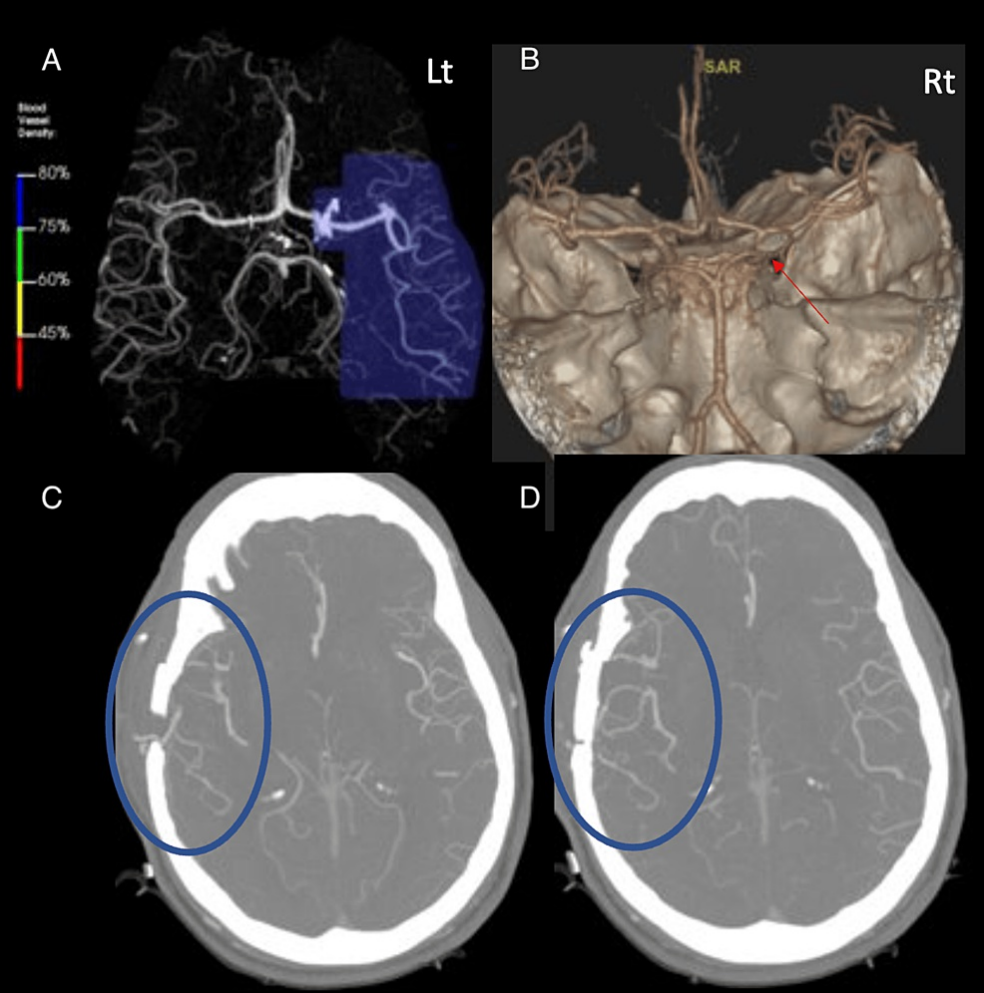
A non-contrast head CT angiogram demonstrating an STA-MCA bypass. Panels A and B depict computerized reconstruction. Panels C and D highlight an area of decreased blood vessel density due to artifact (blue circles). STA: superficial temporal artery, MCA: middle cerebral artery

An STA-MCA (superficial temporal artery to middle cerebral artery) bypass is a surgical procedure used to restore blood flow to the brain’s middle cerebral artery (MCA) by rerouting blood from a scalp artery (STA). This bypass is done to improve blood circulation to the MCA and prevent brain tissue damage due to reduced oxygen delivery. This set of images demonstrates a chronic right internal carotid artery (ICA) occlusion and a patent right superficial temporal artery to middle cerebral artery (STA-MCA) bypass (Figure 4). There is an artifactual appearance of decreased blood vessel density on the left side (blue circles), which is a known phenomenon when the contralateral side experiences increased blood vessel density - in this instance, due to the presence of the STA-MCA bypass. The AI, however, misinterpreted this as a true decreased blood vessel density in the left MCA territory​.

Case 5: subacute bilateral subdural hematomas​ 

**Figure 5:**
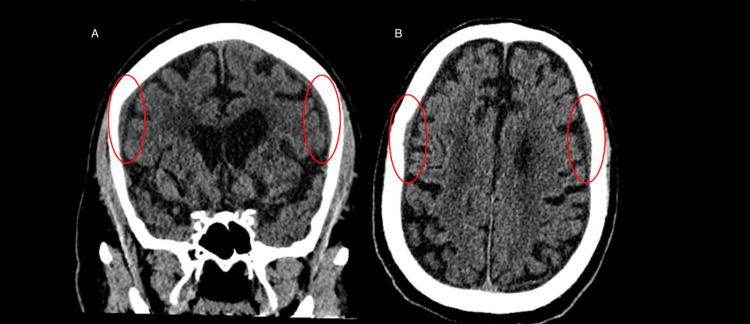
Two images showing a non-contrast head CT demonstrating bilateral subacute subdural hematomas. Panels A and B highlight areas of low-density blood accumulated in the subdural space (red circles).

These images demonstrate bilateral subacute subdural hematomas​ (red circles) (Figure 5). Subdural hematomas occur due to the tearing of bridging cortical veins as they cross the subdural space to drain into an adjacent dural sinus. Subacute hematomas are differentiated from acute hematomas by their distinct gradual accumulation of blood over the course of several days. As the pooled blood undergoes initial degradation and resorption, their appearance on imaging then assumes an intermediate density between that of high-density blood seen in acute hematomas and the low-density blood seen in chronic hematomas. In this case, the AI interpreted this study as normal and failed to detect the subdural hemorrhages.

## Discussion

Case 1: diffuse anoxic ischemic injury

The reason for this AI error is due to the relative decrease of attenuation of the cerebral parenchyma from the diffuse cytotoxic edema of cell death. This results in the relative appearance of increased attenuation of the basal cisterns. Future renditions of the AI software may benefit from tracking the absolute value of the attenuation of the cerebral cortex and the basal cisterns. This means focusing on the actual numerical values representing attenuation rather than the relative differences. By integrating such refinements, the AI could learn to recognize distinct radiological characteristics associated with anoxic injuries and better differentiate them from other pathologies. This case emphasizes the need for AI to deepen its understanding of complex neurological processes using different data modalities to improve its level of diagnostic accuracy. One way this may be achieved is through the use of more advanced machine learning modalities, such as deep reinforcement learning. Deep reinforcement learning is a framework currently being researched that allows for improved accuracy and efficiency in differentiating between conditions that only have subtle distinctions [7].

Case 2: cortical laminar necrosis

This AI error could be attributed to the misinterpretation of hyperattenuation caused by the cortical laminar necrosis in the right parietal and temporal lobes. The algorithm may not have been trained to distinguish between the specific density patterns generated by cortical laminar necrosis and those corresponding to acute subarachnoid hemorrhage. This case highlights the need to improve pattern recognition capabilities of this distinctive gyriform appearance and adjacent relative hypodensity. AI could benefit from additional training with larger datasets and inputs to better distinguish between the two pathologies. This case also serves as an example of the significance of the integration of multi-modal data. The effective diagnosis here was aided by leveraging previous MRI data, which supplied useful information about the restricted diffusion and hyperintensity allowing for a more accurate and comprehensive interpretation by the radiologist. In the context of AI improvement, AI systems could be developed to integrate data from various modalities, such as CT and MRI, to capitalize on the strengths of each to achieve a more accurate and comprehensive diagnosis [8-10].

Case 3: colloid cyst

The reason for this AI error is due to the dense proteinaceous material being misinterpreted as acute blood collection. Although proteinaceous material and acute blood collections may have comparable densities, distinguishing variations do exist. The subtle differences in densities, coupled with the precise location and distinctive well-circumscribed features of colloid cysts, could be employed as distinctive markers. A discrete well-circumscribed area of increased density within the fluid-filled ventricles is unlikely to be hemorrhage, which would be expected to layer or conform to the geometry of the ventricle. The AI’s error in this case emphasizes the significance of not only enhancing AI’s ability to process raw data but also to grasp nuanced variations within different types of pathologies to improve its diagnostic accuracy. It underscores the need to refine its recognition algorithms in specific pathological patterns, such as colloid cysts. AI can be further refined to incorporate more context-aware algorithms that take into consideration the specific location and attributes of various lesions. In addition, as colloid cysts are relatively rare findings, the AI needs to be trained on larger and more diverse training datasets to encompass a wider range of pathologies to improve its accuracy and clinical efficacy across all patient demographics [11,12].

Case 4: right STA-MCA bypass

The reason for this AI error is due to a misinterpretation of the known artifactual phenomenon in the presence of altered vascular flow dynamics from previous surgery or congenital variant anatomy. This demonstrates a need to not only train the AI algorithm on detecting various pathologies but also to expose it to various artifactual peculiarities and post-surgical anatomical variations to augment the algorithm’s accuracy. The failure to recognize this as an artifact could lead to unnecessary interventions that carry risks and can cause harm to patients. In addition, this case underscores the important role that clinical context plays in aiding accurate interpretation. Incorporating patient history, such as past surgical history, can provide the AI with valuable cues to make more accurate and prompt diagnoses [13,14].

Case 5: subacute bilateral subdural hematomas​

This AI error is likely due to the finding that under current models, AI remains relatively insensitive in the detection of subacute and chronic (isodense to hypodense) hemorrhage. ​As AI algorithms typically search for relative hyperdensity when looking for hemorrhages, these potentially life-threatening bleeds can be missed amid the brain parenchyma of similar density. Sensitivity for these findings can be enhanced in future AI algorithms by tuning the program to look for crescentic isodensity along the cerebral convexities and medial displacement of the gyri/sulci. This case highlights the need for AI models with real-time learning capabilities. These AI algorithms can adapt as new input data arise, allowing for the evolution of the AI’s abilities as we learn more about its shortcomings and acquire new imaging objectives [15].

Limitations to the study

This study, while providing valuable insights into the utility of AI in analyzing CT head angiograms, is subject to several limitations that should be acknowledged. First, the sample size of 140 CT head angiograms, although randomly selected, is relatively small. This limitation restricts the generalizability of our findings to larger populations and diverse clinical settings. Furthermore, this study is focused on a single medical center, which may also limit the diversity of the patient population and clinical cases encountered. Finally, there may be a component of human error introduced in the study when the gold standard findings are based on neuroradiologists’ findings, which can be subject to variability in interpretation.

## Conclusions

Overall, it is apparent that AI applications in radiology have much room for improvement before becoming more widely integrated into clinical workflows. Leveraging innovative machine learning algorithms, such as deep reinforcement learning, expands AI's capacity to recognize more complex patterns, improving reliability in discerning conditions with subtle distinctions. Incorporating multimodal data allows for a more definitive interpretation of the visualized pathology as findings from various imaging modalities are cross-referenced and corroborated. In addition, the broader development of AI’s expanding AI's capabilities necessitates that we expose it to larger and more diverse training datasets, thus fostering a more robust understanding of rare pathologies and improving generalizability. Integrating clinical data, such as laboratory results and patient history, can bolster diagnostic accuracy by providing the AI a more complete framework to see and interpret the image through. Furthermore, the ability for real-time learning will allow AI systems to evolve with each case they encounter and new analytical objectives, thus addressing common drawbacks of static AI models. Through the implementation of these developments, radiology AI algorithms can steadily undergo iterative refinement and thus see a progressive increase in diagnostic capabilities, ultimately resulting in enhanced patient prognoses and outcomes. However, despite the promise of AI technology, it is essential to acknowledge the indispensable role of human expertise in the imaging process. It is clear that the integration and synthesis of information by experienced radiologists remain pivotal in arriving at accurate and clinically meaningful diagnoses.
